# NEAT1 is Required for the Expression of the Liver Cancer Stem Cell Marker CD44

**DOI:** 10.3390/ijms21061927

**Published:** 2020-03-11

**Authors:** Shigemi Koyama, Hiroyuki Tsuchiya, Masataka Amisaki, Hiromi Sakaguchi, Soichiro Honjo, Yoshiyuki Fujiwara, Goshi Shiota

**Affiliations:** 1 Division of Molecular and Genetic Medicine, Graduate School of Medicine, Tottori University, 86 Nishi-cho, Yonago 683-8503, Japan; 2 Division of Surgical Oncology, Department of Surgery, Faculty of Medicine, Tottori University, 86 Nishi-cho, Yonago 683-8503, Japan; 3 Division of Radiology, Department of Pathophysiological and Therapeutic Science, Faculty of Medicine, Tottori University, 86 Nishi-cho, Yonago 683-8503, Japan

**Keywords:** cancer stem cells, NEAT1, hepatocellular carcinoma, CD44, spheroid

## Abstract

CD44, a cancer stem cell (CSC) marker, is required for maintaining CSC properties in hepatocellular carcinoma (HCC). Nuclear enriched abundant transcript 1 (NEAT1), a long noncoding RNA (lncRNA), is an oncogenic driver in HCC. In the present study, we investigated the significance of the *NEAT1* gene in association with CD44 expression in liver CSCs of human HCC cell lines. The CSC properties were evaluated by spheroid culture, CSC marker expression, and sensitivity to anti-cancer drugs. The expression of both NEAT1 variant 1 (NEAT1v1) and variant 2 (NEAT1v2) as well as CD44 was significantly increased in the spheroid culture, compared with that in monolayer culture. Overexpression of Neat1v1, but not Neat1v2, enhanced the CSC properties, while knockout of the NEAT1 gene suppressed them. CD44 expression was increased by the overexpression of Neat1v1 and abrogated by NEAT1 knockout. The overexpression of NEAT1v1 restored the CSC properties and CD44 expression in NEAT1-knockout cells. NEAT1v1 expression in HCC tissues was correlated with poor prognosis and CD44 expression. These results suggest that NEAT1v1 is required for CD44 expression. To our surprise, NEAT1v1 also restored the CSC properties even in CD44-deficient cells, suggesting that NEAT1v1 maintains the properties of CSCs in a CD44-independent manner.

## 1. Introduction

Hepatocellular carcinoma (HCC) is one of the leading causes of cancer death worldwide, and is the most prevalent subtype of primary liver cancer, which accounts for about 30,000 deaths each year in Japan [1]. Despite significant progress in HCC treatment, the recurrence rate of HCC is still high, at 60%–70% [2,3]. Therefore, there is a large unmet clinical demand for therapies capable of efficiently curing HCC.

Cancer stem cells (CSCs) are a specific cancer cell subpopulation implicated in initiating and maintaining tumor phenotypes, and are characterized by self-renewal and differentiation potentials, while such properties are not found in the other cancer cells (non-CSCs) [4]. The features of CSCs, such as tumorigenicity and resistance to conventional chemo- and radiotherapy, contribute to their survival, eventually causing recurrence and metastasis, even after these therapies have eliminated non-CSCs [5,6]. Although CSCs were first reported in acute myeloid leukemia [7], various tumors such as those of the brain, breast, lung, colon, and liver have been proven to harbor them [8,9,10,11,12]. In addition, it has been reported that CSCs significantly contribute to development, recurrence, and metastasis in many cancers [5,6]. Therefore, the removal of CSCs is essential to completely eradicate cancer.

Along with the studies highlighting CSC properties, several molecular markers for CSCs have also been identified. CD44, CD133, and epithelial adhesion molecule (EPCAM) are well-known liver CSC markers, and their expression in HCC tissues has been reported to be associated with poor prognosis [12,13,14]. CD44, especially its variant isoform (CD44v), is the most frequently observed CSC marker, such as in gastric, colorectal, breast, and prostate cancers [15,16]. Our previous study suggested that the expression of CD44 standard isoform (CD44s) in HCC tissues is associated with poor prognosis [17]. We also demonstrated that CD44s plays an important role in the maintenance of CSC properties of a human HCC cell line using CD44-knockout (CD44-KO) cells [18]. These findings suggest that CD44 is a promising target for treating HCC.

Nuclear enriched abundant transcript 1 (NEAT1), a long noncoding RNA (lncRNA), is required for the formation of paraspeckles, which are nuclear substructures found in most cultured cells [19]. The NEAT1 gene is expressed as two variant isoforms: NEAT1v1 (3.8 kb in length in humans) and NEAT1v2 (22.7 kb). Both of these are transcribed from the same nucleotide position, but have different sites of transcriptional termination [19]. Although the function of paraspeckles remains unclear, it was reported that NEAT1 induces IL8 transcription in response to immune stimuli [20]. Moreover, NEAT1 is also involved in the development of cancer by suppressing p53 function, or by forming a transcriptional repressor complex [21,22,23]. However, it has been reported that NEAT1 has tumor-suppressive function under the transcriptional control of p53 [24]. In Japanese patients with HCC, the mutation rate of the *NEAT1* gene was significantly higher than other lncRNAs [25], suggesting that NEAT1 is an important lncRNA in HCC.

In the present study, we investigated the function of NEAT1 in liver CSCs. Our findings revealed that NEAT1, especially NEAT1v1, plays a critical role in the maintenance of CSC properties of HCC cell lines. Moreover, we demonstrated that NEAT1v1 is required for CD44 expression. Intriguingly, NEAT1 restored CSC properties in CD44-KO cells. Given the malignant potential of CD44 in HCC, these results suggest that NEAT1 is involved in the development and progression of HCC in both CD44-dependent and -independent manners.

## 2. Results

### 2.1. Increased Expression of NEAT1 in Spheroids

**Figure 1:**
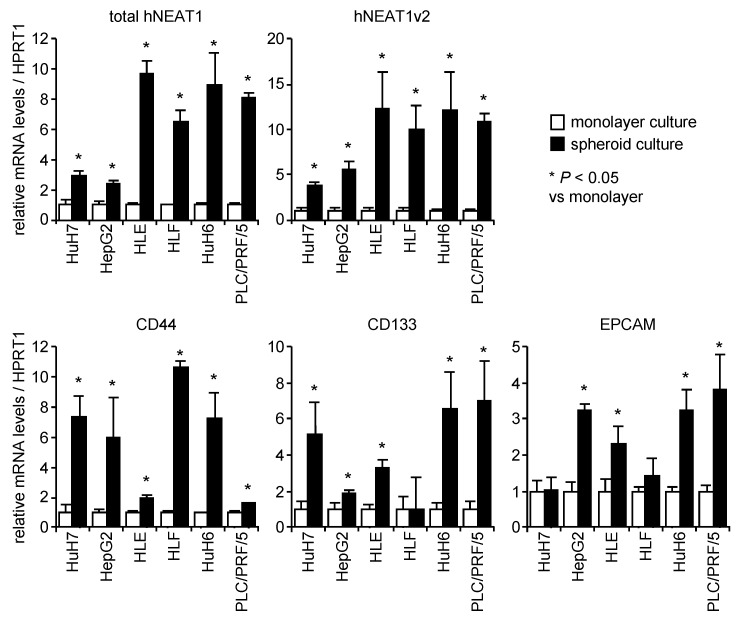
mRNA expression levels of CSC markers relative to HPRT1 in monolayer cells (white columns) and in spheroids (black columns) (*n* = 3). Data were normalized to the expression levels in the monolayer cells of each cell line. * *p* < 0.05 vs. monolayer cells; Student’s *t*-test.

HCC cell lines efficiently formed spheroids after 7 days of incubation in the spheroid medium (Figure S1A). Because CSCs possess the ability to perform anchorage-independent growth, the spheroid culture led to the enrichment of CSCs, as indicated by the increased expression of CSC markers including CD44, CD133, and EPCAM (Figure 1). This observation might be a consequence of the selective growth of CSCs under the anchorage independent culture condition as previously observed [26,27]. It is noteworthy that these CSC markers showed a cell line-preferential expression pattern while the expression levels of NEAT1 were almost similar among the cell lines (Figure S1B). In contrast, the expression of NEAT1v1 and NEAT1v2 was concomitantly increased in spheroids of all HCC cell lines tested (Figure 1 and Figure S1B). These results indicate that NEAT1 expression is upregulated in CSCs.

### 2.2. Increased Spheroid Formation and CD44 Expression by Mouse Neat1v1

**Figure 2:**
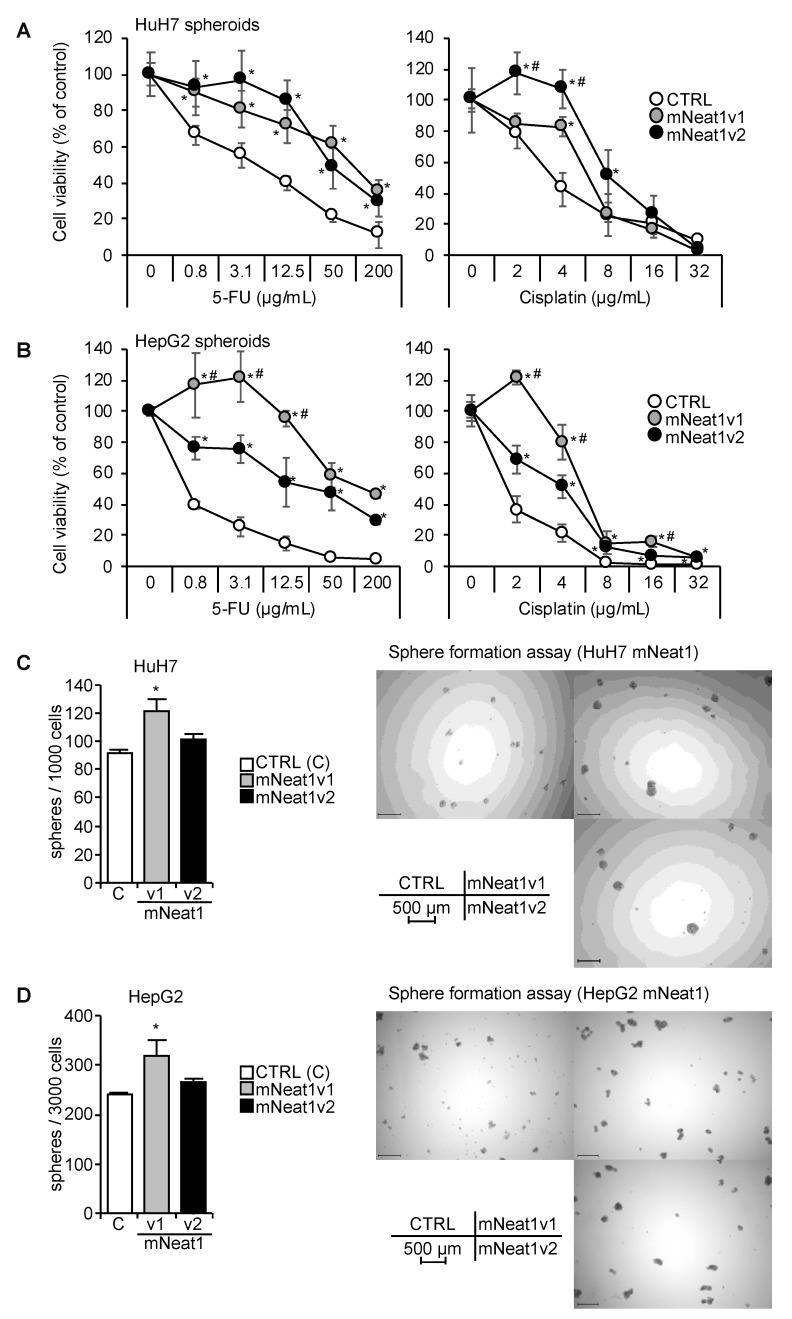
Increased CSC properties by mNeat1v1. (**A**,**B**) Viability of spheroids of HuH7 (**A**) or HepG2 (**B**) cells in the presence of 5-FU or cisplatin. White circles, mock-transfected cells (CTRL), gray circles, mNeat1v1-expressing cells; black circles, mNeat1v2-expressing cells. * *p* < 0.05 vs. CTRL; # *p* < 0.05 mNeat1v1 vs. mNeat1v2, Turkey’s test (*n* = 4). (**C**,**D**) Spheroid formation ability of HuH7 (**C**) and HepG2 (**D**) cells. White columns, mock-transfected cells (CTRL), gray columns, mNeat1v1-expressing cells; black columns, mNeat1v2-expressing cells. * *p* < 0.05 vs. CTRL; Dunnett’s test (*n* = 4).

In HuH7 and HepG2 cells, the induction rate of NEAT1 expression in spheroids were lower than the other cell lines (Figure 1). Moreover, HuH7 and HepG2 cells harbor mutant and wild-type TP53 gene, respectively, which is suggested to be related to the function of NEAT1 [21,22,23,24]. Thus, hereafter, we used these two cell lines in further experiments. Although the sequence conservation of NEAT1 is not very high between human and mouse (Figure S2A), they share significant structural similarity, in which NEAT1v2 forms an intramolecular RNA-RNA interaction between its 5′ and 3′ regions [28]. Thus, we produced HuH7 and HepG2 cells stably overexpressing mNeat1v1 or mNeat1v2 (Figure S2B). A poly(A) tail is added to mNeat1v1 while it is not found in mNea1v2, which allows us to discriminate between mNeat1v1 and mNeat1v2. Thus, we designed a mNeat1v1-specific oligo(dT) reverse-transcription primer (mV1-dT14) (Figure S2C). The expression levels of mNeat1v2 using mV1-dT14 in mNeat1v2-overexpressing HuH7 and HepG2 cells were decreased by less than 10%, compared to those using random nanomer primer (N9), suggesting that the non-specific reverse-transcription of mNeat1v2 by mV1-dT14 is almost negligible. The expression levels of total mNeat1 in mNeat1v1-overexpressing cells did not markedly change between N9 and mV1-dT14 (Figure S2C), suggesting that mV1-dT14 reverse-transcribes mNeat1v1 as efficiently as N9 does. Whereas, total mNeat1 expression levels using mV1-dT14 in mNeat1v2-overexpressing cells were decreased by approximately 60% (HuH7) or 70% (HepG2), compared to those using N9 (Figure S2C). These results suggest that the expression levels of mNeat1v1 in mNeat1v2-overexpressing cells are approximately 1.5 (HuH7)- or 2.3 (HepG2)-fold higher than those of mNeat1v2. Spheroids of mNeat1-overexpressing cells showed resistance to 5-FU and cisplatin (Figure 2A,B). However, the number of spheroids was significantly higher only in mNeat1v1 transformants than in the control, and mNeat1v2 transformants did not show a significant increase in spheroid formation (Figure 2C,D).

**Figure 3:**
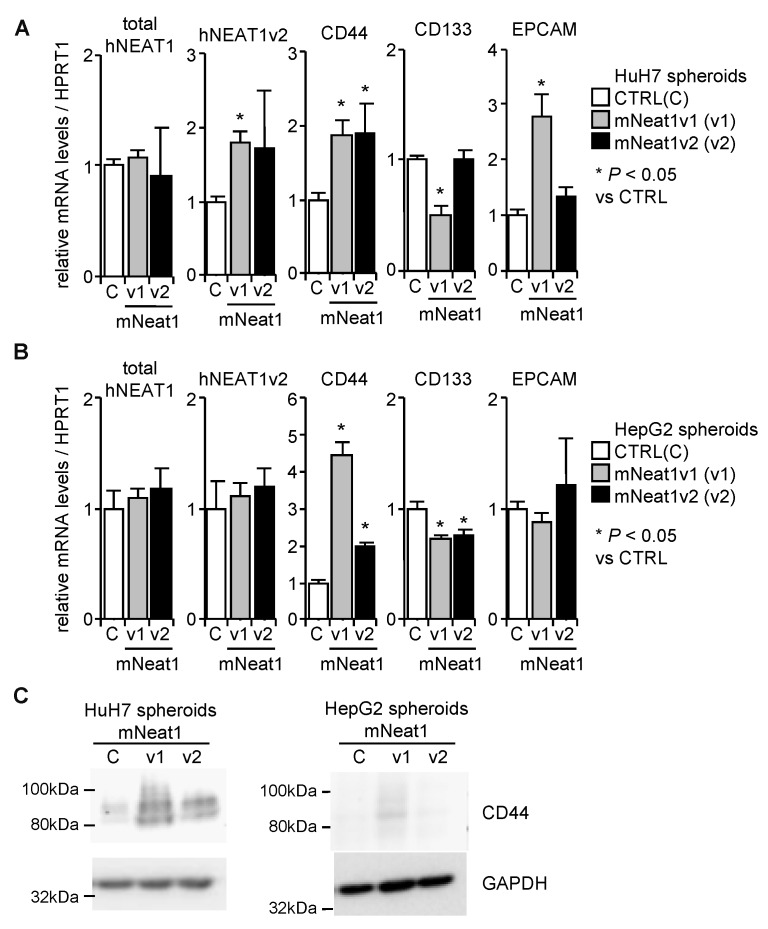
Increased CD44 expression in spheroids by mNeat1v1. (**A**,**B**) mRNA expression levels relative to HPRT1 in spheroids of HuH7 (**A**) and HepG2 (**B**) cells (*n* = 3). White columns, mock-transfected cells (CTRL, **C**); gray columns, mNeat1v1-expressing cells (v1); black columns, mNeat1v2-expressing cells (v2). * *p* < 0.05 vs. CTRL spheroids; Dunnett’s test. (**C**) CD44 protein expression in spheroids of HuH7 (left) and HepG2 (right)-expressing mock (**C**), mNeat1v1 (v1), or mNeat1v2 (v2). GAPDH was used as an internal control.

The expression of hNEAT1 in spheroids was not significantly affected by mNeat1, except for NEAT1v2 in HuH7 cells, which was significantly upregulated by mNeat1v1 (Figure 3A,B). The expression of CD44 in spheroids of both cell lines was significantly upregulated by mNeat1 (Figure 3A,B). CD133 was significantly decreased by mNeat1v1 in HuH7 and HepG2 cells, and by mNeat1v2 in HepG2 cells, but was not changed by mNeat1v2 overexpression in HuH7 cells (Figure 3A,B). EPCAM was significantly upregulated only in HuH7 cells overexpressing mNeat1v1 (Figure 3A,B). The expression of CD44 protein was markedly increased by mNeat1v1 (Figure 3C). These results suggest that mNeat1v1 maintains liver CSCs and induces CD44 expression.

### 2.3. Decreased Spheroid Formation and CD44 Expression by NEAT1 Knockout

**Figure 4:**
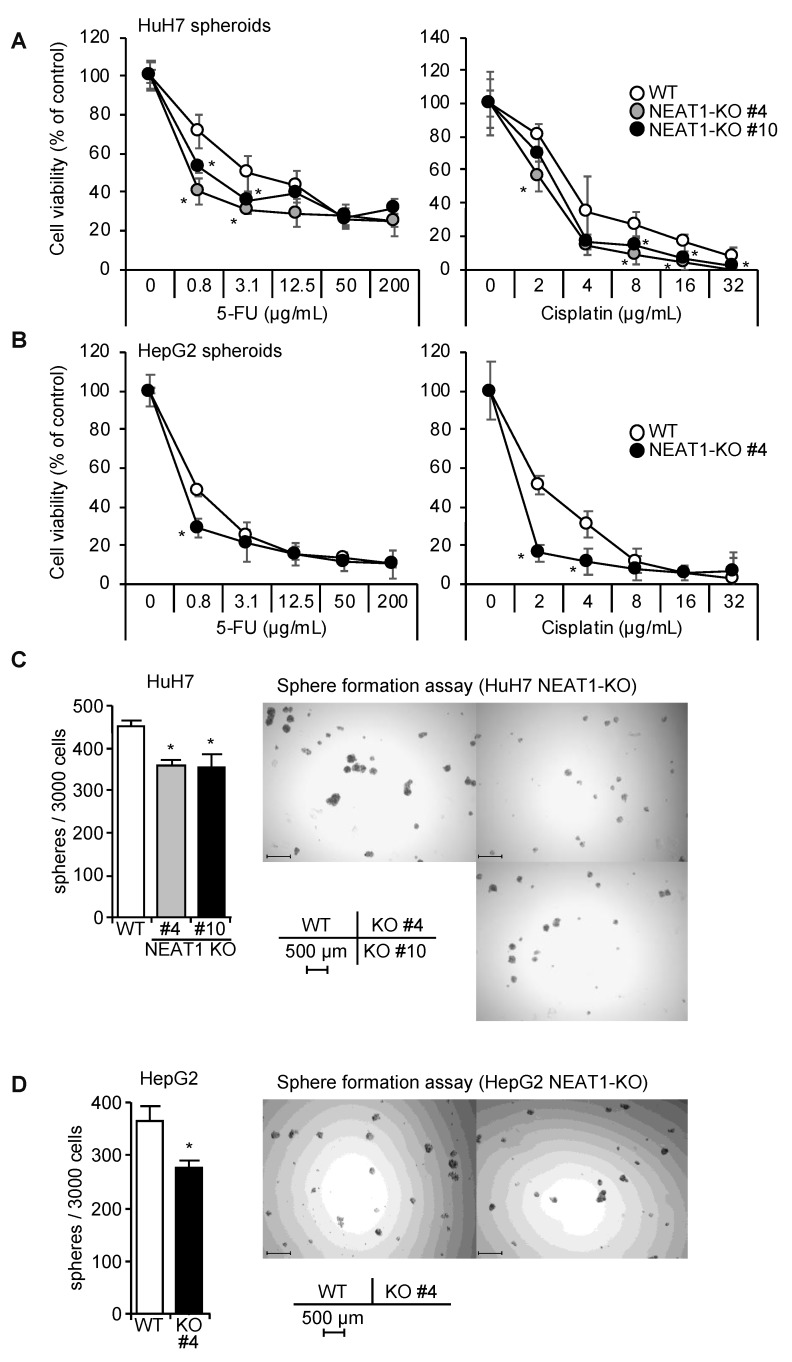
Decreased CSC properties by NEAT1 deficiency. (**A**,**B**) Viability of spheroids of HuH7 (**A**) or HepG2 (**B**) cells in the presence of 5-FU or cisplatin. White circles, parental cells (WT), gray and black circles, NEAT1-KO cells. * *p* < 0.05 vs. CTRL; # *p* < 0.05 NEAT1-KO #4 vs. #10, Turkey’s test (HuH7) or Student’s *t*-test (HepG2) (*n* = 4). (**C**,**D**) Spheroid formation ability of HuH7 (**C**) and HepG2 (**D**) cells. White columns, parental cells (WT); gray and black columns, NEAT1-KO cells. * *p* < 0.05 vs. WT; Dunnett’s test (HuH7) or Student’s *t*-test (HepG2) (*n* = 4).

By employing the CRISPR/Cas9 system, NEAT1-KO clones were established (#4 and #10 in HuH7, which were created by sgNEAT1a and sgNEAT1b, respectively; #4 in HepG2, created by sgNEAT1b) (Figure S3). Spheroids of NEAT1-KO HuH7 and HepG2 cells showed increased sensitivity to 5-FU and cisplatin (Figure 4A,B). Spheroid formation abilities of both cell lines were significantly decreased by NEAT1 deficiency (Figure 4C,D).

**Figure 5:**
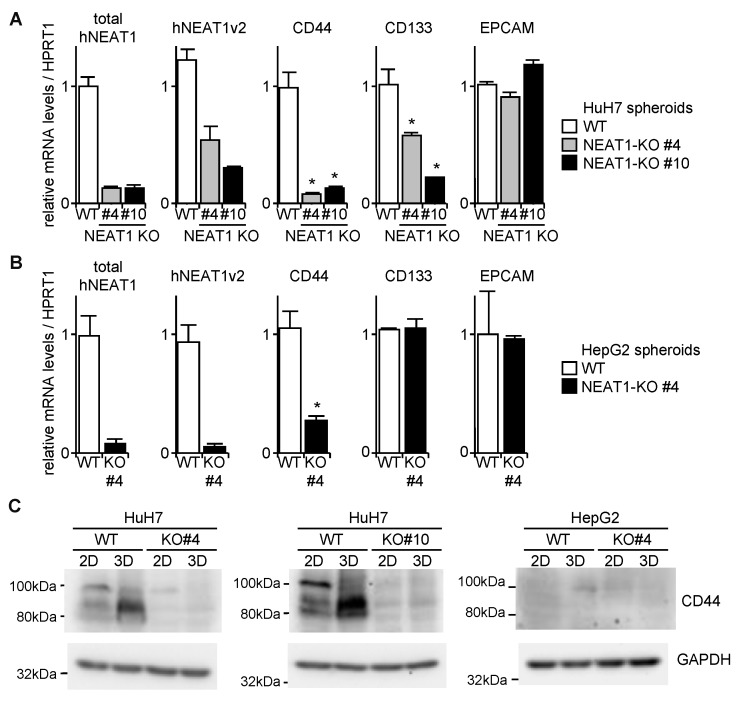
Decreased CD44 expression in spheroids by NEAT1 deficiency. (**A**,**B**) mRNA expression levels relative to HPRT1 in spheroids of HuH7 (**A**) and HepG2 (**B**) cells. White columns, parental cells (WT); gray and black columns, NEAT1-KO cells. * *p* < 0.05 vs. WT; Dunnett’s test (HuH7) or Student’s *t*-test (HepG2) (*n* = 3). (**C**) CD44 protein expression in monolayer cells (2D) and in spheroids (3D) of HuH7 (left and middle) and HepG2 (right). WT, parental cells; KO, NEAT1-KO cells. GAPDH was used as an internal control.

CD44 mRNA expression in spheroids of NEAT1-KO was markedly decreased, while CD133 was significantly decreased by NEAT1 deficiency in HuH7 cells, and was not changed in HepG2 cells (Figure 5A,B). EPCAM was not affected by NEAT1 deficiency in both cell lines (Figure 5A,B). In agreement with these findings, the expression of CD44 protein was abrogated in NEAT1-KO HuH7 cells (Figure 5C). In HepG2 cells, because CD44 protein expression was not observed even in WT cells, no effects of NEAT1 deficiency on CD44 expression were observed (Figure 5C). These results suggest that hNEAT1 is involved in the regulation of CSCs, and is also required for CD44 expression.

### 2.4. Increased Expression of p21^CDKN1A^ in NEAT1-KO Cells

P21^CDKN1A^ is a target gene of p53, while NEAT1 modulates p53 function [21,24]. In the spheroids of HuH7 and HepG2 cells, mNeat1v1 clearly downregulated p21^CDKN1A^ (Figure S4A). In contrast, NEAT-KO spheroids showed increased p21^CDKN1A^ (Figure S4B). However, although the knockdown of p21^CDKN1A^ by siRNA significantly increased the number of spheroids of WT cells, it did not rescue the spheroid-forming ability of NEAT1-KO cells (Figure S4C,D). Moreover, NEAT1 deficiency did not affect the cell cycle distribution in monolayer and spheroids (Figure S5A,B). These results suggest that NEAT1 suppresses p21^CDKN1A^ expression in HCC cells, but it does not have marked effects on CSCs.

### 2.5. NEAT1v1-Induced CD44 Expression

**Figure 6:**
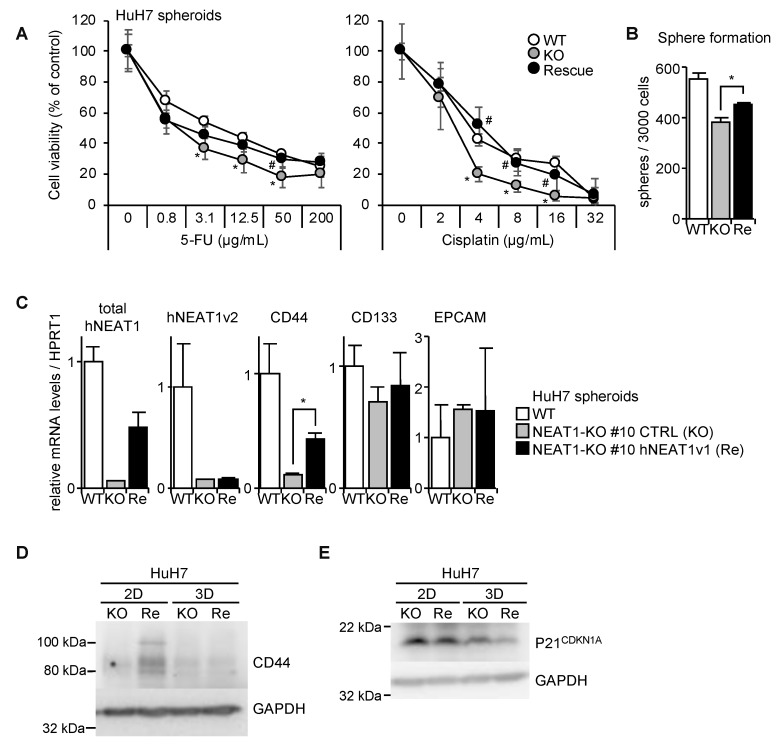
Phenotype rescue of NEAT1 deficiency in spheroids by hNEAT1v1 overexpression. (**A**) Viability of spheroids of HuH7 (white circles, WT), HuH7 NEAT1-KO #10 cells expressing mock (gray circles, KO) or hNEAT1v1 (black circles, Rescue) in the presence of 5-FU (left) or cisplatin (right). * *p* < 0.05 vs. WT; # *p* < 0.05 KO vs. Re, Turkey’s test (*n* = 4). (**B**) Spheroid formation ability of HuH7 cells (white columns, WT), and HuH7 NEAT1-KO #10 cells expressing mock (gray column, KO) or hNEAT1v1 (black column, Re). * *p* < 0.05, KO vs. Re; Tukey–Kramer’s test (*n* = 4). (**C**) mRNA expression levels relative to HPRT1 in spheroids of parental HuH7 cells (white columns, WT), and HuH7 NEAT1-KO #10 cells expressing mock (gray columns, KO) or hNEAT1v1 (black columns, Re). * *p* < 0.05, KO spheroids vs. Re spheroids; Tukey–Kramer’s test. (*n* = 3–4). (**D**,**E**) CD44 (**D**) and P21^CDKN1A^ (**E**) protein expressions in monolayer cells (2D) and in spheroids (3D) of HuH7 NEAT1-KO #10 cells expressing mock (KO) or hNEAT1v1 (Re). GAPDH was used as an internal control.

To clarify whether NEAT1 expression rescues the CSC phenotype, hNEAT1v1-expressing vector was stably transfected into HuH7 NEAT1-KO #10 cells. The overexpression of hNEAT1v1 conferred 5-FU and cisplatin resistance, and rescued the spheroid-forming ability (Figure 6A,B). CD44 expression was also partially rescued (Figure 6C,D). CD133 and EPCAM expressions were not markedly changed by hNEAT1v1 overexpression (Figure 6C). In addition, hNEAT1v1 suppressed p21^CDKN1A^ expression (Figure 6E). These results suggest that hNEAT1v1 enhances CSC properties, and that CD44 and p21^CDKN1A^ are potential target genes of hNEAT1v1 in HCC cells.

### 2.6. Rescue of CD44-KO Cells by NEAT1v1

**Figure 7:**
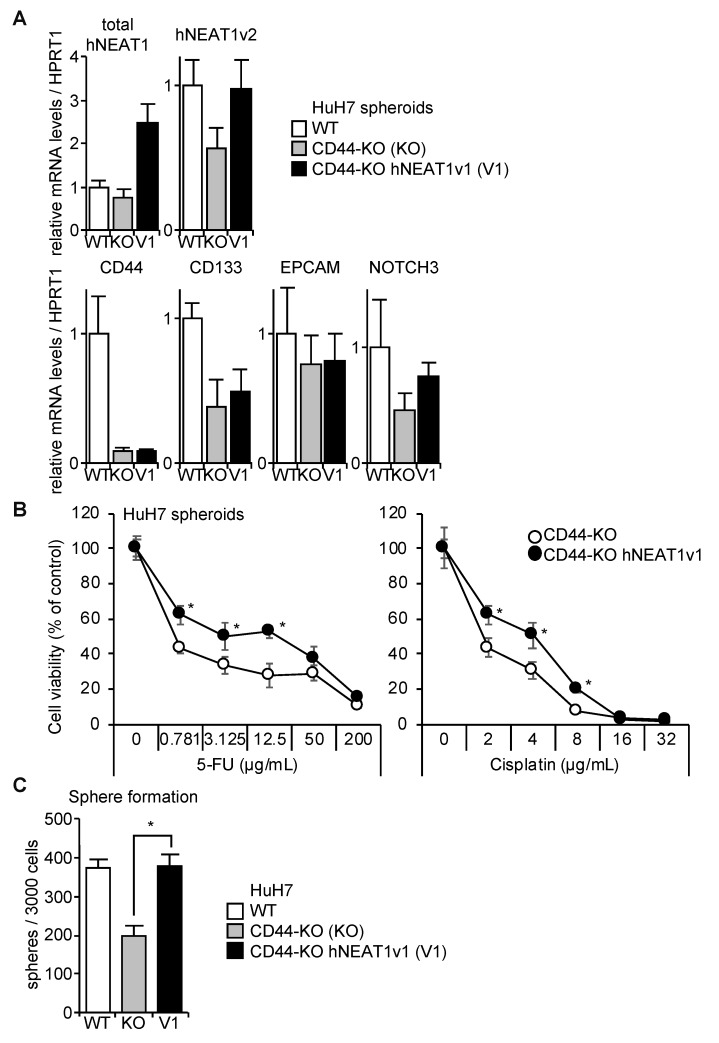
Phenotype rescue of CD44 deficiency in spheroids by hNEAT1v1 overexpression. (**A**) mRNA expression levels relative to HPRT1 in spheroids of parental HuH7 cells (white columns, WT), and CD44-KO cells expressing mock (gray columns, KO) or hNEAT1v1 (black columns, V1) (*n* = 3). (**B**) Viability of spheroids of HuH7 CD44-KO cells expressing mock (white circles, CTRL) or hNEAT1v1 (black circles) in the presence of 5-FU (left) or cisplatin (right). * *p* < 0.05 vs. CTRL; Student’s *t*-test (*n* = 4). (**C**) Spheroid formation ability of parental HuH7 cells (white columns, WT) and CD44-KO cells expressing mock (gray columns, KO) or hNEAT1v1 (black columns, V1). * *p* < 0.05, KO vs. hNEAT1v1; Student’s *t*-test (*n* = 4).

We previously reported that CD44 maintains CSC properties possibly through NOTCH3 in HCC cells [18]. Because CD44 expression is induced by hNEAT1v1, it was investigated whether hNEAT1v1 regulates CSC properties via CD44. The overexpression of hNEAT1v1 rescued the expression of NEAT1 in CD44-KO cells while CSC markers were not changed by hNEAT1v1 (Figure 7A). The overexpression of hNEAT1v1 conferred 5-FU and cisplatin resistance, and, to our surprise, rescued the spheroid-forming ability of CD44-KO cells (Figure 7B,C). These results suggest that hNEAT1v1 regulates the CSC properties of HCC in a CD44-independent manner. In agreement with this notion, the expression of NOTCH3, which was decreased in spheroids of CD44-KO cells compared with those of parental cells, was not affected by hNEAT1v1 (Figure 7A).

### 2.7. Association of NEAT1v1 with Poor Prognosis in Patients with HCC

Using our in-house cohort (Table S1) [18], we determined the expression levels of total NEAT1 and NEAT1v2 in HCC patients. However, these expression levels did not show statistically significant differences between HCC tumors and their surrounding non-tumor tissues (Figure S6A). Moreover, no effects of NEAT1 expression levels in HCC tumors on overall survival and recurrence-free survival periods were observed (Figure S6B). To discriminate NEAT1v1 from NEAT1v2, the patients were divided into two groups, total NEAT1 dominant and NEAT1v2 dominant, by a regression line between total NEAT1 and NEAT1v2 (Figure S6A). Because dominant expression of total NEAT1 over NEAT1v2 is expected as a consequence of increased expression of NEAT1v1, we considered the patients with the dominant expression of total NEAT1 in HCC tumors as NEATd1v1 dominant. This stratification revealed that high total NEAT1 expression, according to the median as a cut-off value, was associated with poor recurrence-free survival in NEAT1v1 dominant patients (*p* = 0.0457, Figure S6C). A similar trend was observed in patients with high NEAT1v2 expression in the NEAT1v1 dominant group (*p* = 0.100, Figure S6C). In contrast, in the NEAT1v2 dominant group, patients with high total NEAT1 expression tended to have better overall survival than patients with low total NEAT1 expression (*p* = 0.166, Figure S6D). All other analyses of prognosis did not show apparent differences by NEAT1 expression (Figure S6C,D).

### 2.8. Association of NEAT1v1 with Poor Prognosis in Patients with HCC

We previously reported [18] that CD44 expression was significantly upregulated in HCC tumor tissues of the same cohort (Table S1). Regression plots revealed that CD44 expression was significantly correlated with total NEAT1, while no significant correlation was observed between CD44 and NEAT1v2 (Figure S7A). The same correlations were also found in NEAT1v1 dominant and NEA1v2 dominant groups (Figure S7B,C), suggesting that NEAT1v1 is involved in CD44 expression in HCC.

## 3. Discussion

In the present study, we demonstrated that NEAT1 is a lncRNA required for CD44 expression, and plays a critical role in maintaining CSC properties of HCC cell lines. We overexpressed mNeat1 in HCC cell lines although the identity of human and mouse NEAT1 sequences is not highly conserved (Figure S2A). However, both human and mouse NEAT1 RNAs share significant similarity in higher order structure [28]. In addition, KO and rescue experiments with hNEAT1v1 clearly demonstrated that NEAT1v1 is sufficient for CD44 expression and CSC regulation in human HCC cell lines. In addition, the overexpression of NEAT1v1 enhanced CSC properties in CD44-KO cells, suggesting that NEAT1 regulates CSC properties in a CD44-independent manner. It was also shown that p21^CDKN1A^ has a suppressive effect on spheroid formation of HCC cells, while NEAT1v1 suppresses p21^CDKN1A^ expression. However, NEAT1 deficiency did not affect the cell cycle of HCC cells, and knockdown of p21^CDKN1A^ expression did not rescue spheroid formation in NEAT1-KO cells. These results suggest that p21^CDKN1A^ is a negative target gene of NEAT1v1, but is not involved in the function of NEAT1v1 in liver CSCs.

CSCs have several malignant characteristics, including tumor-initiating ability, chemo- and radio-resistance, and high metastatic potential. Anchorage-independent growth also represents the stemness of cancer. Spheroid culture is an in vitro surrogate method to assess the ability to self-renew, and has been utilized to concentrate CSCs in preclinical studies [26,29,30]. Under our spheroid culture conditions, HCC cell lines showed spheroids that were asymmetric and round or distorted in appearance (Figure S1). These spheroids showed apparently increased expression of CSC markers, including CD44, CD133, and EPCAM, compared with monolayer cultured cells (Figure 1). These results indicate the successful enrichment of CSCs by the spheroid culture. Although the upregulation of CD133 and EPCAM in HLF cells and EPCAM in HuH7 was not observed, the expression of CD44 was significantly increased in all HCC cell lines, suggesting that CD44 plays a central role in liver CSCs. It was demonstrated that NEAT1 did not have marked effects on the expression of the other major CSC markers, CD133 and EPCAM. Although CD44^+^CD133^+^ cells in HCC cell lines showed more potent CSC properties than single positive cells, CD44^+^ HCC cells have superior invasion ability while clonogenic growth ability is more prominent in CD133^+^ HCC cells [14,31]. It was also demonstrated that EPCAM expression in HCC was associated with hepatic stem cell/hepatoblast makers as well as CD133 [12]. These results suggest that CD44 and NEAT1 regulate different aspects of CSC properties from CD133 and EPCAM.

CD44 is a membrane protein functioning as a receptor for glycosaminoglycans, especially for hyaluronan, in various tissues [15,32]. It is well known that the *CD44* gene expresses various transcript variants, among which the shortest one called standard variant (CD44s) has been shown to regulate liver CSCs [18]. In HuH7 and mNeat1v1-overexpressing HepG2 cells, the expression of CD44s was observed, while other splicing variants were not detected. It has been reported that CD44 expression in tumor cells is regulated by transcription factors and miRNAs [33,34,35,36,37]. A lncRNA, gastric adenocarcinoma predictive long intergenic noncoding RNA (GAPLINC), also induces CD44 expression by suppressing miR-221, a CD44-targeting miRNA [33]. Although the suppression of miR-221 by NEAT1 has not been reported, a number of miRNAs, including miR-101, miR-124, miR-129, miR-193a, miR-612, and miR-613, are reportedly regulated by NEAT1 in HCC [22,38,39,40,41,42].

In the present study, although hNEAT1v1 overexpression induces CD44 mRNA expression in spheroids, CD44 protein expression was observed only in the rescue cells in the monolayer culture condition (Figure 6D). It was reported that the deubiquitylation of CD44s protein by USP28 is required for maintaining the invasive and metastatic phenotypes of CSCs in human bladder cancer [43]. In HCC, c-MYC requires USP28 for its protein stabilization [44], implying that USP28 may also function in liver CSCs. However, NEAT1v1 maintains CSCs properties in a CD44-independent manner, suggesting that NEAT1v1 activates pathways other than USP28. Therefore, there is a possibility, although further extensive studies are certainly required, that the inconsistent expression of CD44 protein might be due to the post-translational regulation of CD44.

*NEAT1* has been recognized as a p53 target gene [21,22,23,24]. NEAT1 harbors a consensus p53-binding site in its promoter region, and p53, upon its activation under stress conditions, such as DNA damage, induces NEAT1 transcription [21,24]. Adriaens et al. [21] demonstrated that p53-induced NEAT1 activates the ATR signaling to alleviate DNA damage in preneoplastic cells undergoing replication stress. In this context, NEAT1 allows the cells to overcome oncogene-induced replication stress, eventually leading to tumorigenesis. Yang et al. [22] demonstrated that NEAT1 increases the proliferation of HCC cells by suppressing p53 expression and activating cyclin D1 expression. In contrast, although Mellow et al. [24] also found that *NEAT1* is a p53 target gene, they showed that NEAT1 protects cells from oncogenic transformation. Interestingly, NEAT1 deficiency did not affect doxorubicin-induced cell cycle arrest and apoptosis, while oncogenic transformation was increased [24]. Another group also showed that the suppression of p53-induced NEAT1 expression impaired the tumor-suppressive functions of p53 [45]. It has recently been reported by the same group that high NEAT1 expression resulted in significant extension of the overall survival period only in patients with wild type p53 [46]. Based on this observation, they concluded that NEAT1 exerts its tumor-suppressive function in a manner dependent on p53 status [46]. Interestingly, they also found that the expression levels of NEAT1 in tumor tissues compared with those in normal tissues were increased, decreased, or constant depending on the original sites of tumors, and were significantly increased even in p53 mutant tumor tissues [46]. In the present study, we mainly employed HuH7 (p53 mutant) and HepG2 (p53 wild type) cells, both of which showed the suppressive effect of NEAT1 on p21^CDKN1A^ expression. The results indicate that complex regulatory mechanisms, in which not only p53 but also other factors unrelated to p53 are involved, underlie the functions and expression of NEAT1.

In the present study based on in vitro experiments, we demonstrated that NEAT1v1 is sufficient to increase spheroid formation. However, further studies including NEAT1v2-specific knockdown are required to determine whether NEAT1v2 is involved in the regulation of CSC properties. Because these variants have the same transcription start site, but their transcriptions terminate at different sites without splicing, NEAT1v1 harbors the same sequence as part of NEAT1v2. However, human and mouse NEAT1v1s, but not NEAT1v2s, have a poly(A) tail. Taking advantage of this fact, we designed a mNeat1v1-specific oligo(dT) reverse transcription primer (mV1-dT14), and successfully discriminated mNeat1v1 expression from mNeat1v2 expression. We also designed hNEAT1v1-specific oligo(dT) reverse transcription primer (hV1-dT14) to determine the expression levels of hNEAT1v1 in HCC cells. However, this primer failed to specifically reverse-transcribe hNEAT1v1 as the apparent non-specific amplification of hNEAT1v2 was detected. This might be because human NEAT1v2 (NR_131012) has five consecutive A repeats (10 to 22 repeats) [46]. Whereas, mouse Neat1v2 (NR_131212) does not have such repeats. Therefore, the discrimination of NEAT1v1 from NEAT1v2 with oligo(dT) primers is still challenging in human cells, requiring complicated optimization of the reverse transcription reaction condition. Thus, it is technically difficult to precisely discriminate the expression level of NEAT1v1 from that of NEAT1v2 by RT-qPCR and RNA-seq. This might be one of the causes of the contradictory reports on the function of NEAT1 [21,24]. The stratification of our cohort [18] by a regression line between total NEAT1 and NEAT1v2 demonstrated the apparent association of NEAT1 expression with poor prognosis in patients with NEAT1v1 dominant expression. In contrast, in the NEAT1v2 dominant group, total NEAT1 expression tended to be associated with good prognosis. These observations may reflect that NEAT1v1 and NEAT1v2 have contradictory or at least different functions in HCC. Therefore, it is worthwhile pursuing the precise determination of NEAT1v1 expression in tumors.

However, it is still unclear why CSC properties in mNeat1v2-overexpressing cells were not increased. One possible explanation is that NEAT1v2 may suppress NEAT1v1 function in liver CSCs. NEAT1v2 is required for paraspeckle formation, into which NEAT1v1 is also incorporated, while NEAT1v1 also exists in “microspeckle”, outside of paraspeckles [47], suggesting that NEAT1v1 has intrinsic functions independent of NEAT1v2. However, structural analyses of NEAT1 revealed that NEAT1v2 forms an intramolecular RNA-RNA interaction between its 5’ and 3’ regions [28]. Because the 5’ region of NEAT1v2 overlaps NEAT1v1, it is suggested that NEAT1v1 interacts with the 3’ region of NEAT1v2. This interaction may pull NEAT1v1 into paraspeckles, and inhibit its microspeckle localization. Moreover, NEAT1v1 expression levels are considered more abundant (five-fold or more) than NEAT1v2 in many cells and tissues [19,28,48]. This may provide an advantage for the independent biological function of NEAT1v1. In contrast, the expression levels of mNeat1v1 in our mNeat1v2-overexpressing cells is only 1.5- to 2.3-fold higher than mNeat1v2. Therefore, the insufficient mNeat1v1 expression, relative to mNeat1v2, might be the cause of this discrepancy. However, NEAT1v2-specific knockdown experiments are still necessary to precisely assess the roles of NEAT1v2 in the regulation of CSC properties.

In conclusion, NEAT1v1 maintains CSC properties in HCC cell lines in a CD44-independent manner. Given the fact that CD44s has an important role in HCC, NEAT1v1 would be a potential therapeutic target for HCC associated with high CD44 expression. On the other hand, the precise mechanisms, especially the target genes of NEAT1v1, required for the maintenance of CSC properties would also be promising targets for liver CSCs.

## 4. Materials and Methods

### 4.1. Cell Culture

Human hepatocellular carcinoma HuH7, HepG2, HLE, HLF, HuH6, and PLC/PRF/5, and human immortalized fibroblast KMST-6 were purchased from the Japanese Collection of Research Bioresources Cell Bank (Osaka, Japan), and were maintained in DMEM (Nissui Pharmaceutical, Tokyo, Japan) supplemented with 10% inactivated FBS (Sigma-Aldrich, St. Louis, MO, USA).

Spheroid culture was previously reported [18]. In brief, cells were plated in an ultra-low-attachment 24-well plate (Corning, Corning, NY, USA) at 1000–3000 cells/well in Ham’s F-12 medium (Nacalai, Kyoto, Japan) containing 20 ng/mL recombinant human epidermal growth factor (PeproTech, Rocky Hill, NJ, USA), 20 ng/mL recombinant human basic fibroblast growth factor (PeproTech), 1 × B27 (Thermo Fisher Scientific, Waltham, MA, USA), and 0.52% methylcellulose (Sigma-Aldrich). The cells were cultured for 7 days to form spheroids. The number of spheroids was counted by ImageJ software (Bethesda, MD, USA).

### 4.2. Reverse-Transcription Quantitative PCR (RT-qPCR)

Following recovery of total RNA with Sepasol-RNA I Super G (Nacalai), complementary DNA was reverse-transcribed with reverse transcription primers (Table S2) by ReverTra Ace (Toyobo, Otsu, Japan) according to the manufacturer’s instructions. The human and mouse NEAT1v1-specific reverse transcription primers were designed based on the PolyA-seq data (GSE30198) [49], which revealed a major poly(A) site of NEAT11v1 in human and mouse livers. qPCR was performed on ViiA 7 Real-time PCR System (Thermo Fisher Scientific) by using THUNDERBIRD SYBR qPCR Mix (Toyobo) with primers shown in Supplementary Table S2. β-actin was used as an internal control for calculation of relative mRNA expression levels. For comparison of monolayer cells and spheroids, hypoxanthine phosphoribosyltransferase 1 (HPRT1) was used as an internal control instead of β-actin, whose expression level is readily affected by the culture conditions.

### 4.3. Western Blot Analysis

Monolayer cells and spheroids were washed with phosphate-buffered saline and lysed in 150 µL of RIPA buffer (50 mM Tris-HCl (pH 7.9), 150 mM NaCl, 1% NP-40, 0.5% sodium deoxycholate, 0.1% sodium dodecyl sulfate (SDS)). After freezing the lysate at −80°C, the protein concentration was determined by the BCA assay (Nacalai), and was adjusted with the RIPA buffer followed by mixing with 2× Laemmli sample buffer (10% β-mercaptoethanol, 125 mM Tris-HCl (pH 6.8), 4% SDS, 20% glycerol, 0.004% bromophenol blue). The samples were subjected to SDS-polyacrylamide gel electrophoresis. Following transfer to the PVDF membranes, immunoblotting was performed. Antibodies against CD44 (#3570; Cell Signaling Technology, Danvers, MA, USA), glyceraldehyde-3-phosphate dehydrogenase (GAPDH) (sc-365062; Santa Cruz Biotechnology, Santa Cruz, CA, USA), and p21^CDKN1A^ (sc-397-G; Santa Cruz Biotechnology) were used.

### 4.4. Stable Transfection of NEAT1-Expressing Vectors

Mouse Neat1v1 (mNeat1v1)- and Neat1v2 (mNeat1v2)-expressing plasmids, pCMV-mNeat1v1 and pCMV-mNeat1v2, respectively, were kindly provided by Dr. Shinichi Nakagawa [19,20]. pAcGFP-C1 (Clontech, Mountain View, CA, USA) digested with *Mlu*I was ligated into the plasmids, resulting in pCMV-mNeat1v1-AcGFP and pCMV-mNeat1v2-AcGFP. pCRII_TOPO_hNEAT1 harboring human NEAT1v1 was a gift from Dr. Archa Fox (#61518; Addgene, Cambridge, MA, USA) [50]. The fragment of pCRII_TOPO_hNEAT1 digested with *Bam*HI and *Xba*I was ligated into the *Nhe*I and *Bam*HI sites of pcDNA6/V5-His B (Thermo Fisher Scientific), resulting in pcDNA6-hNEAT1v1. pAcGFP-N1 (Clontech) was digested with *Bam*HI and *Bgl*II, and self-ligated. The resultant plasmid was digested with *Afl*II, and ligated with AflII-linker oligoDNA (Suppementary Table S2) to create a *Bam*HI site. The resultant plasmid digested with *Bam*HI and pcDNA6-hNEAT1v1 digested with *Bgl*II were ligated, resulting in pcDNA6-hNEAT1v1-AcGFP. pCMV-mNeat1v1-AcGFP, pCMV-mNeat1v2-AcGFP, or pcDNA6-hNEAT1v1-AcGFP were transfected into cells with LipofectAMINE2000 (Thermo Fisher Scientific). Following G418 or blasticidin (Blst) selection, GFP-positive cells were sorted by flow cytometry.

### 4.5. Knockout of the NEAT1 Gene

Single-guide RNAs targeting the human *NEAT1* gene (sgNEAT1a and sgNEAT1b) are shown in Supplementary Table S2. Double-stranded oligo DNAs harboring the sequences were ligated into the *Bbs*I sites of pSpCas9(BB)-2A-GFP (PX458) (Addgene, #48138) [51], resulting in psgNEAT1a and psgNEAT1b, respectively, which express both single-guide RNA and SpCas9. A knock-in vector for the human *NEAT1* gene was constructed on a plasmid, DT-A/AFP-pA/PGK-Neo-pA, which was kindly provided by Dr. Yasuhide Furuta (RIKEN Center for Life Science Technologies, Kobe, Japan: http://www2.clst.riken.jp/arg/cassette.html). This plasmid and pmCherry-N1 (Clontech) were digested by *Sal*I and *Bsr*GI and ligated to replace the EGFP gene in the DT-A/AFP-pA/PGK-Neo-pA with the mCherry gene, resulting in pDTApA-mCherry. Here, 5′- and 3′-homology arms were amplified with the primers shown in Supplementary Table S2, and KOD-plus-NEO (Toyobo) using genomic DNA from KMST-6 cells as a template. The PCR products were inserted into *Afl*II and *Sac*II, and *Xba*I and *Xho*I sites in pDTApA-mCherry, resulting in pTarget-hNEAT1. The nucleotide sequence was confirmed by the dideoxy method.

pTarget-hNEAT1 together with psgNEAT1a or psgNEAT1b was transfected into HuH7 and HepG2 cells with LipofectAMINE2000. Following G418 selection, genomic DNA was recovered from several clones expressing the mCherry gene and subjected to PCR with knock-in check primers (Supplementary Table S2). HuH7 cells in which the NEAT1 gene was knocked out by sgNEAT1a and sgNEAT1b are designated as NEAT1-KO #4 and #10, respectively. HepG2 cells in which the NEAT1 gene was knocked out by sgNEAT1b are designated as NEAT1-KO #4. Their parental cells are referred to as WT where relevant.

### 4.6. SiRNA Transfection

Validated p21^CDKN1A^-specific siRNAs (si1 and si2) (Silencer Select siRNA s417 and s415, respectively) and negative control siRNA (siNC) (Silencer Select Negative Control No. 2 siRNA) were purchased from Thermo Fisher Scientific, and were transfected into cells with Lipofectamine RNAiMax (Thermo Fisher Scientific). Western blotting for the validation of p21^CDKN1A^ knockdown was performed 2 days after the transfection. Spheroid culture was started 1 day after siRNA transfection.

### 4.7. Cell viability Assay

Spheroids were seeded into a ultra-low-attachment 96-well plate (Corning), and were treated with 0–200 µg/mL 5-fluorouracil (5-FU) (Nacalai) or 0–32 µg/mL cisplatin (Sigma-Aldrich) for 72 h. The concentration of solvent (dimethyl sulfoxide) was kept at 0.1%. Cell Counting Kit-8 (Dojindo, Kumamoto, Japan) was used to determine cell viability, in accordance with the manufacturer’s protocol. Absorbances at 450 and 600 nm were measured using a plate reader.

### 4.8. Analysis of Human Samples

The HCC patient samples used here have been previously reported [18]. This study was approved by the Institutional Review Board of our institution in accordance with the ethical standards laid down in the 1964 Declaration of Helsinki and its later amendments (Institutional Review Board approval number: 18A071). Liver specimens from 92 HCC patients (Table S1) were obtained at Tottori University Hospital between 2004 and 2013 and immediately stored in RNAlater (Qiagen, Valencia, CA, USA) at −80 °C. RNA from frozen specimens was recovered and purified using TRIzol reagent and RNeasy Plus Kit (QIAGEN), in accordance with the manufacturer’s instructions. Complementary DNA synthesis and RT-qPCR were performed as described above. Medical records were reviewed retrospectively to collect the clinical data of the patients.

### 4.9. Statistical Analysis

Three or more independent samples for each experiment were analyzed, and values were shown in mean ± standard deviation. Student’s *t*-test was performed to assess the differences between two groups. Multiple comparisons were made by Dunnett’s test or Tukey–Kramer’s test. A *p* value less than 0.05 was considered as statistically significant.

## Supplementary Materials

Supplementary materials can be found at https://www.mdpi.com/1422-0067/21/6/1927/s1.

## Abbreviations

5-FU	5-Fluorouracil
Blst	Blasticidin
CD44v	CD44 variant isoform
CD44s	CD44 standard isoform
CSC	Cancer stem cell
EPCAM	Epithelial adhesion molecule
GAPDH	Glyceraldehyde-3-phosphate dehydrogenase
HCC	Hepatocellular carcinoma
HPRT1	Hypoxanthine phosphoribosyltransferase 1
KO	Knock out
lncRNA	Long noncoding RNA
miR	MicroRNA
NEAT1	Nuclear enriched abundant transcript 1
PTBP3	Polypyrimidine tract-binding proteins 3
RT-qPCR	Reverse-transcription quantitative PCR
SD	Standard deviation
