# Supplementary material for: NEAT1 is Required for the Expression of the Liver Cancer Stem Cell Marker CD44
Source: Int J Mol Sci. 2020 Mar 11;21(6):1927. doi: 10.3390/ijms21061927 (PMC7139689; doi:10.3390/ijms21061927)
Supplement: Supplementary file 1 [file ijms-21-01927-s001.zip › 6.SupFiles_R1/SupFigs_R1.pdf]

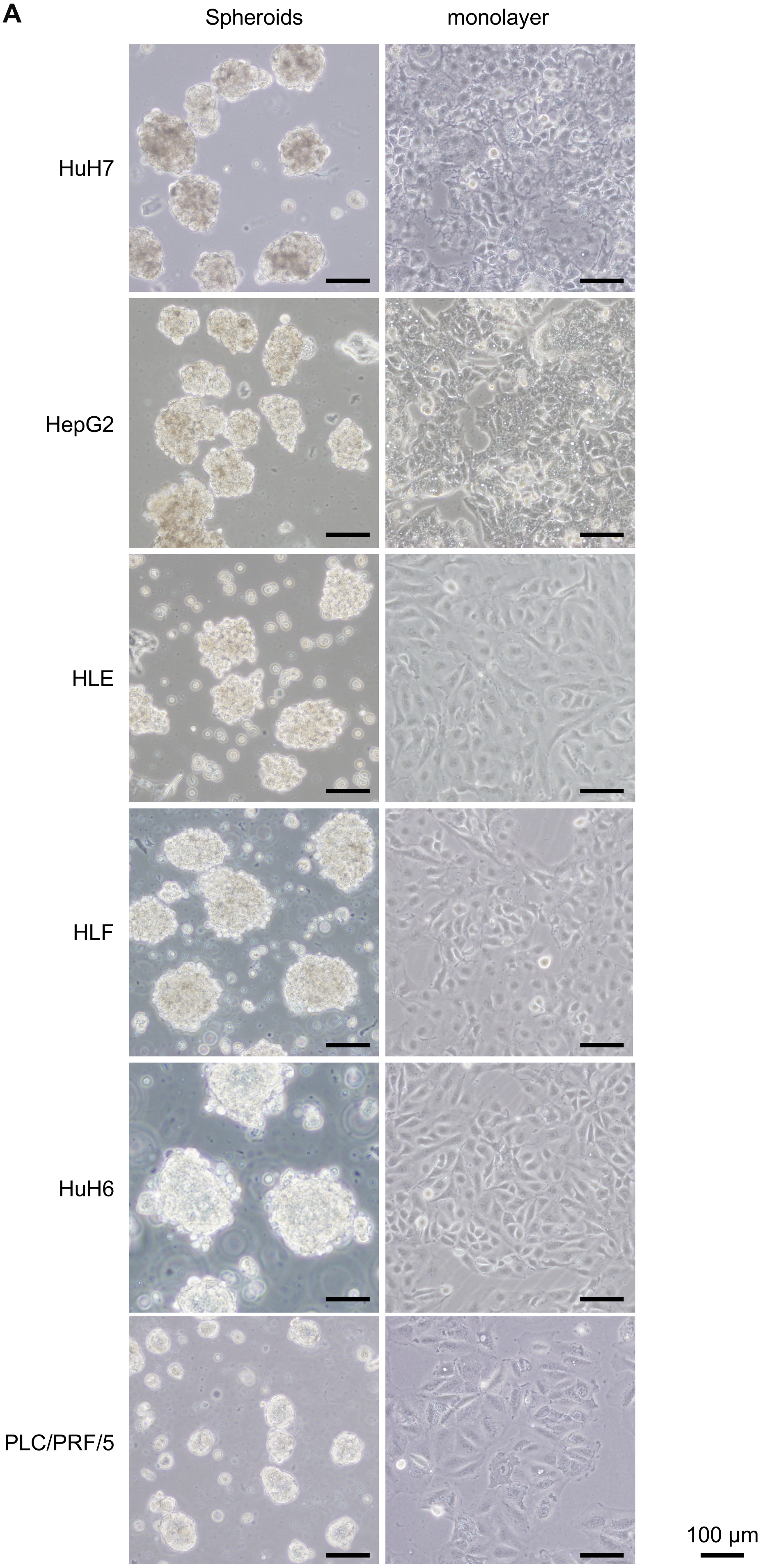

Figure S1 Spheroid culture of HCC cell lines. **A,** Representative images of spheroids of HCC cell lines.

(Figure S1, continue)

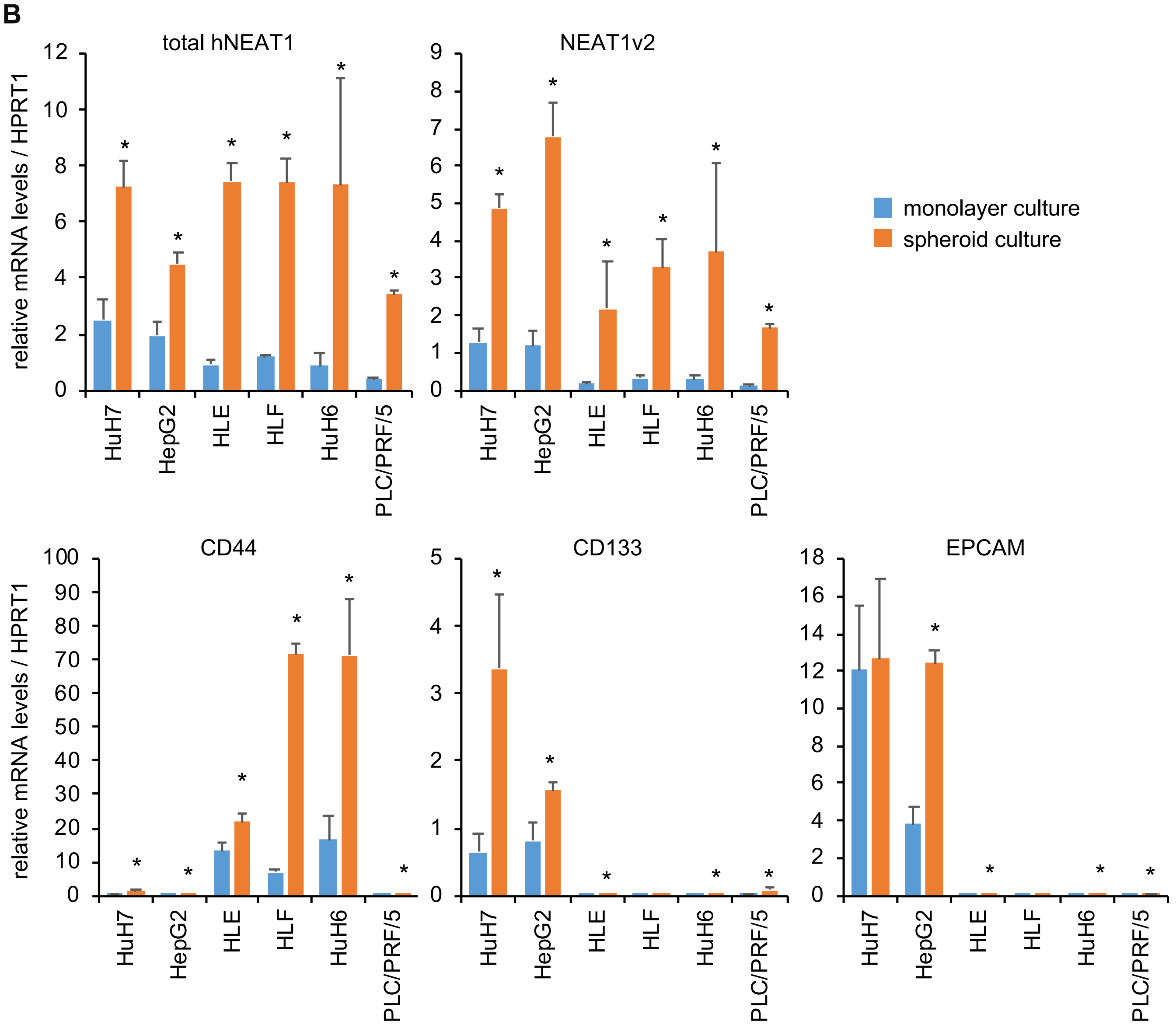

(Figure S1, continue) **B**, mRNA expression levels of CSC markers relative to HPRT1 in monolayer cells (blue columns) and in spheroids (orange columns) ($n = 3$). The data are the same as those shown in **Figure 1**, but without normalization to the expression levels in the monolayer cells.

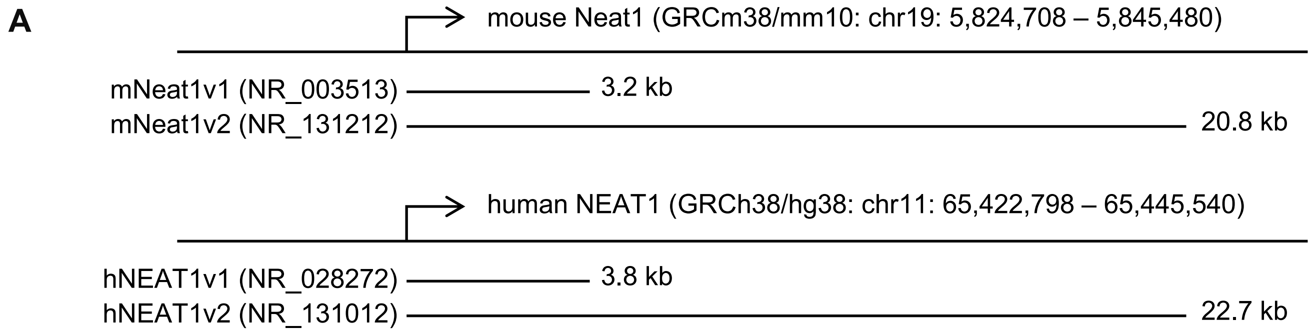

	percent identity (hNEAT1/mNeat1)	
**Alignment tools**	**variant 1**	**variant 2**
BLASTN	69%	69%
MAFFT	65.3%	55.6%
Kalign	64.4%	53.8%
Clustal Omega	55.2%	55.5%

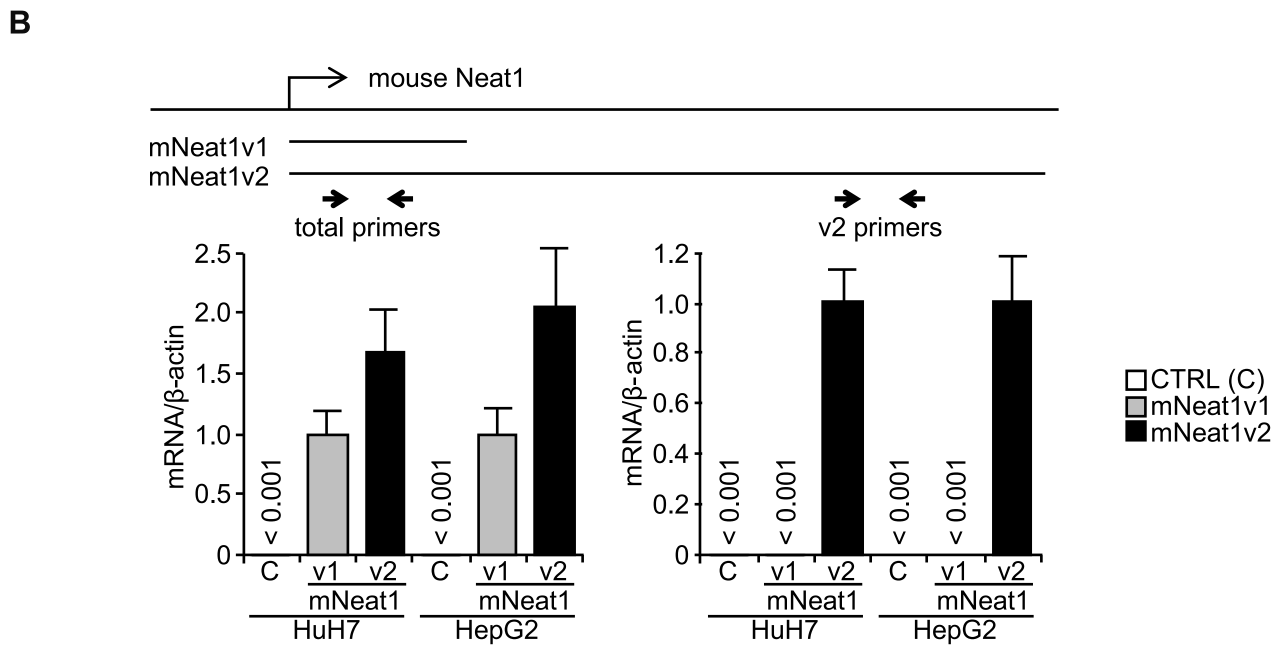

Figure S2 Establishment of HCC cell lines expressing mouse Neat1 variant 1 (mNeat1v1) and Neat1 variant 2 (mNeat1v2). **A**, Genomic structure of mouse (upper) and human (lower) NEAT1. Identity of the mouse and human *NEAT1* gene is summarized in the table. hNEAT1v1, human NEAT1 variant 1; hNEAT1v2, human NEAT1 variant 2. **B**, The expression levels of mNeat1 in HCC cells stably transfected with mock (*open columns*), mNeat1v1 (*gray columns*) or mNeat1v2 (*black columns*) plasmids. Total primers (*left*) amplify both mNeat1v1 and mNeat1v2, while v2 primers (*right*) amplify only mNeat1v2. Their locations are shown. ($n = 4$).

(Figure S2, continue)

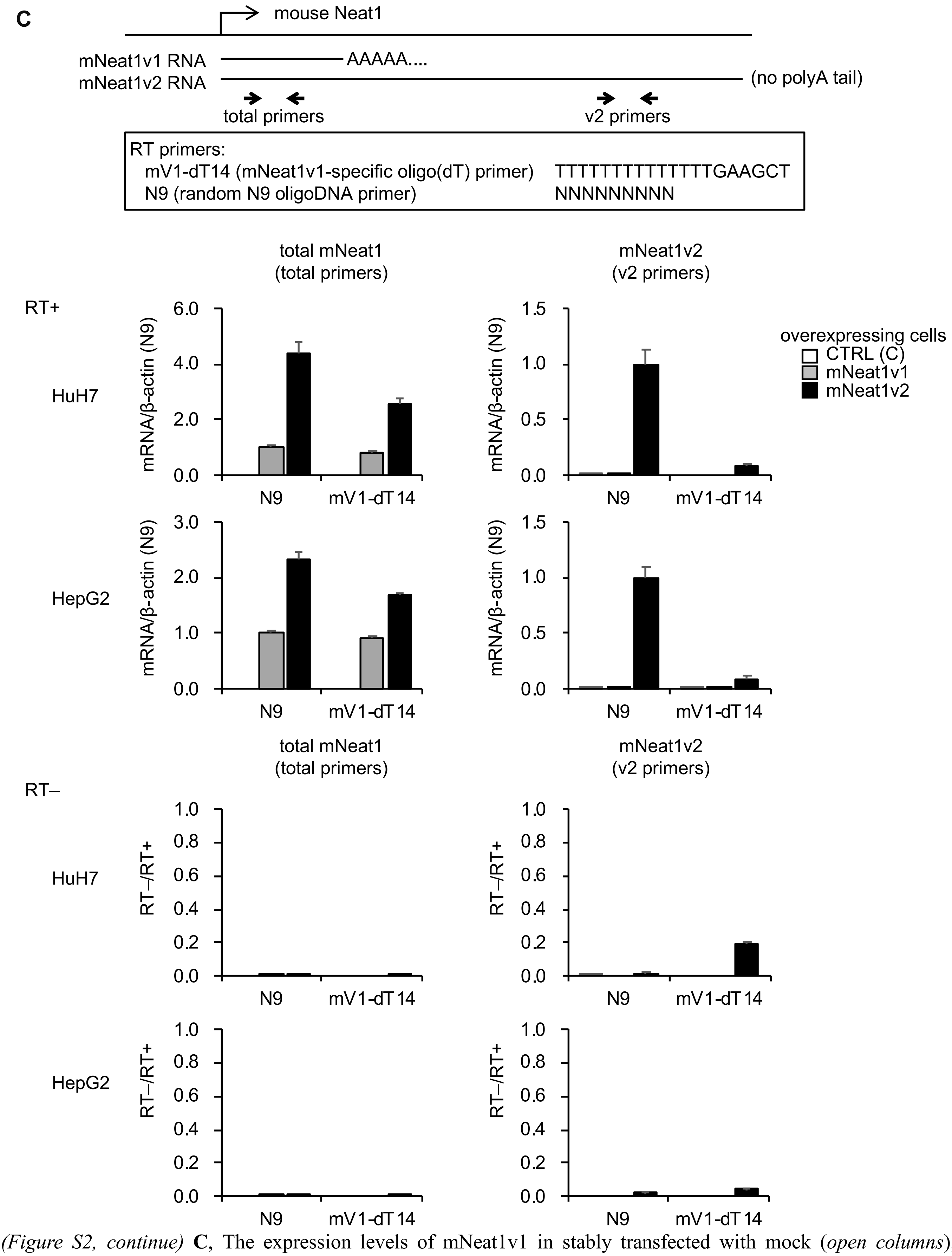

(Figure S2, continue) **C**, The expression levels of mNeat1v1 in stably transfected with mock (*open columns*), mNeat1v1 (*gray columns*) or mNeat1v2 (*black columns*) plasmids. Following DNase treatment, the total RNA were reverse-transcribed with mNeat1v1-specific oligo(dT) (mV1-dT14) or random N9 oligoDNA (N9) primers by ReverTra Ace (TOYOBO, Japan) (RT+). The data sown as RT+ were normalized by the expression levels of β-actin that was reverse-transcribed by the N9 primer. RT–; total RNA incubated in the same reaction mixture without ReverTra Ace. The ratio of RT– to RT+ was shown to indicate the negligible contamination of genomic DNA. (n = 4).

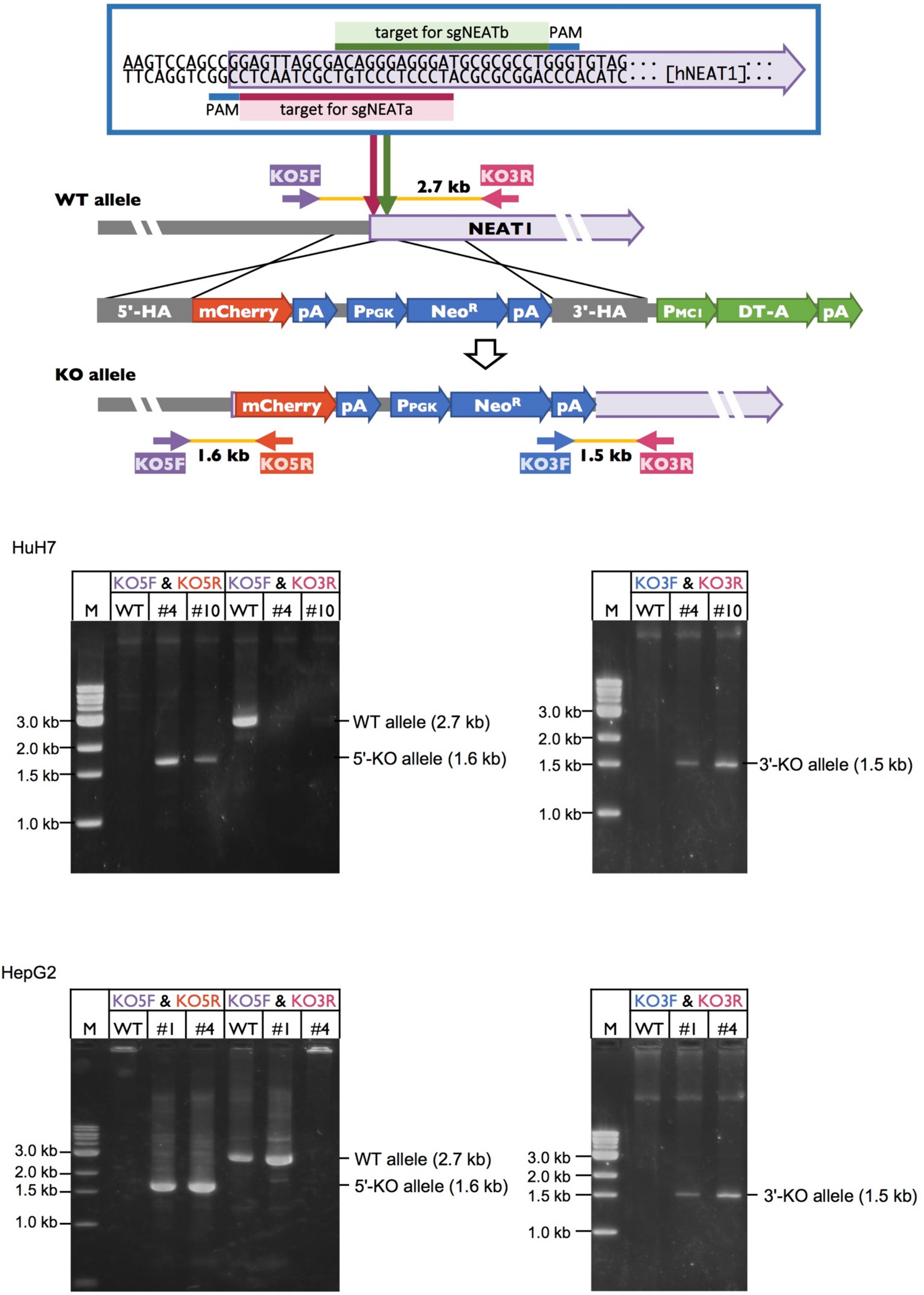

Figure S3 Establishment of NEAT1-KO HCC cell lines. We used two different sgRNAs (sgNEATa and sgNEATb), which were designed to target sites proximal to the transcription start site of the *NEAT1* gene. Genomic DNA PCR with primers indicated in this figure demonstrated that both alleles were deleted in HuH7 clone #4 obtained by sgNEATa and clone #10 obtained by sgNEAT1b, and HepG2 clone #4 obtained by sgNEATb. HepG2 clone #1 had single allele deletion.

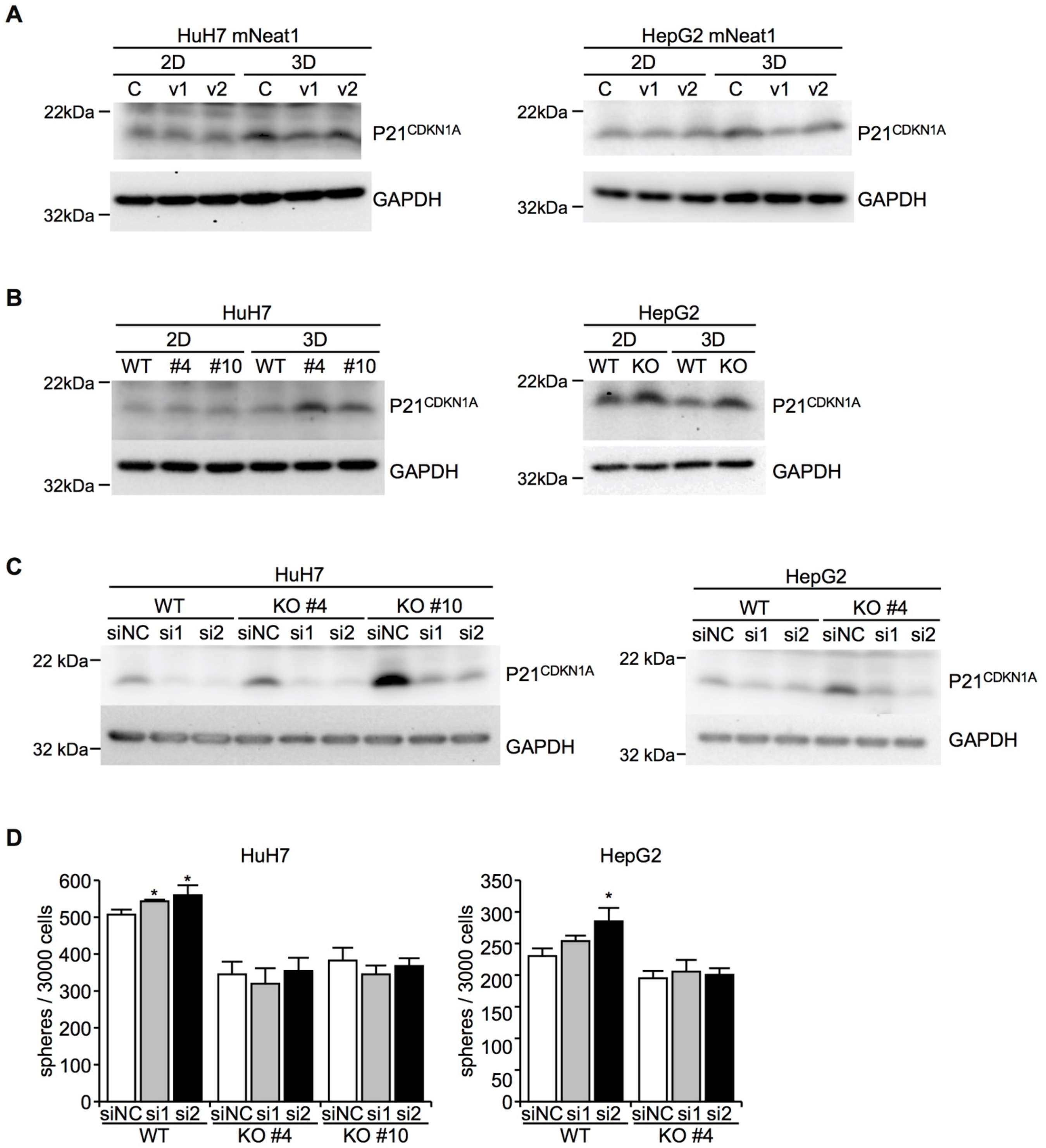

Figure S4 Involvement of P21^{CDKN1A} in spheroid formation of HCC cell lines. **A**, P21^{CDKN1A} expression in HCC cell lines expressing mNeat1v1 (v1) and mNeat1v 2 (v2). **B**, P21^{CDKN1A} expression in NEAT1-KO HCC cell lines. **C**, Confirmation of P21^{CDKN1A} knockdown in HCC cell lines. GAPDH was used as an internal control. **D**, Spheroid formation ability of HCC cell lines transfected with a negative control siRNA (siNC) or siRNAs targeting P21^{CDKN1A} (si1 and si2). *$P < 0.05$ vs. siNC; Dunnett's test ($n = 4$).

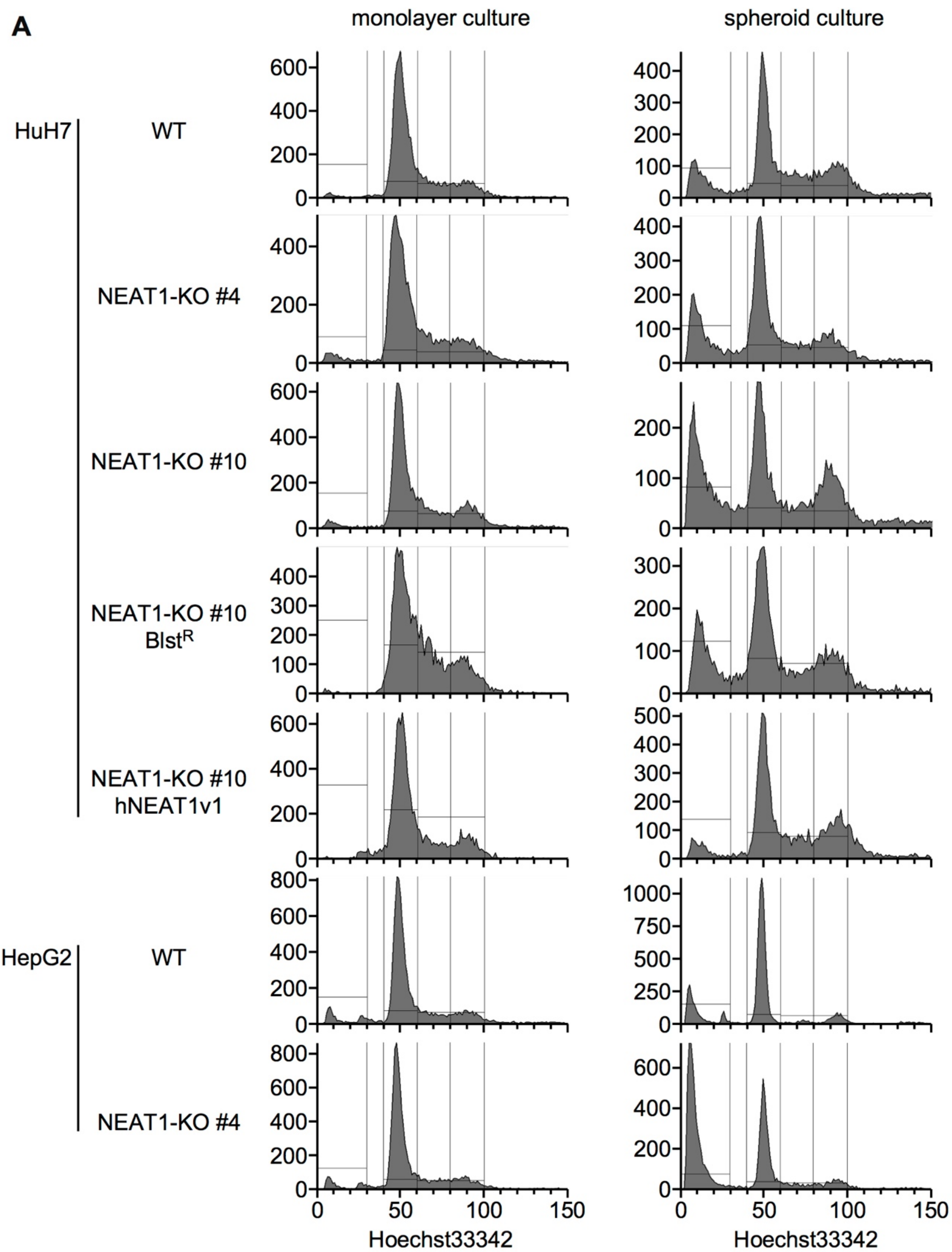

Figure S5 Cell cycle analysis of NEAT1-KO HCC cell lines. **A**, Representative histograms of flow cytometry analysis of NEAT1-KO cell lines.

(Figure S5, continue)

B

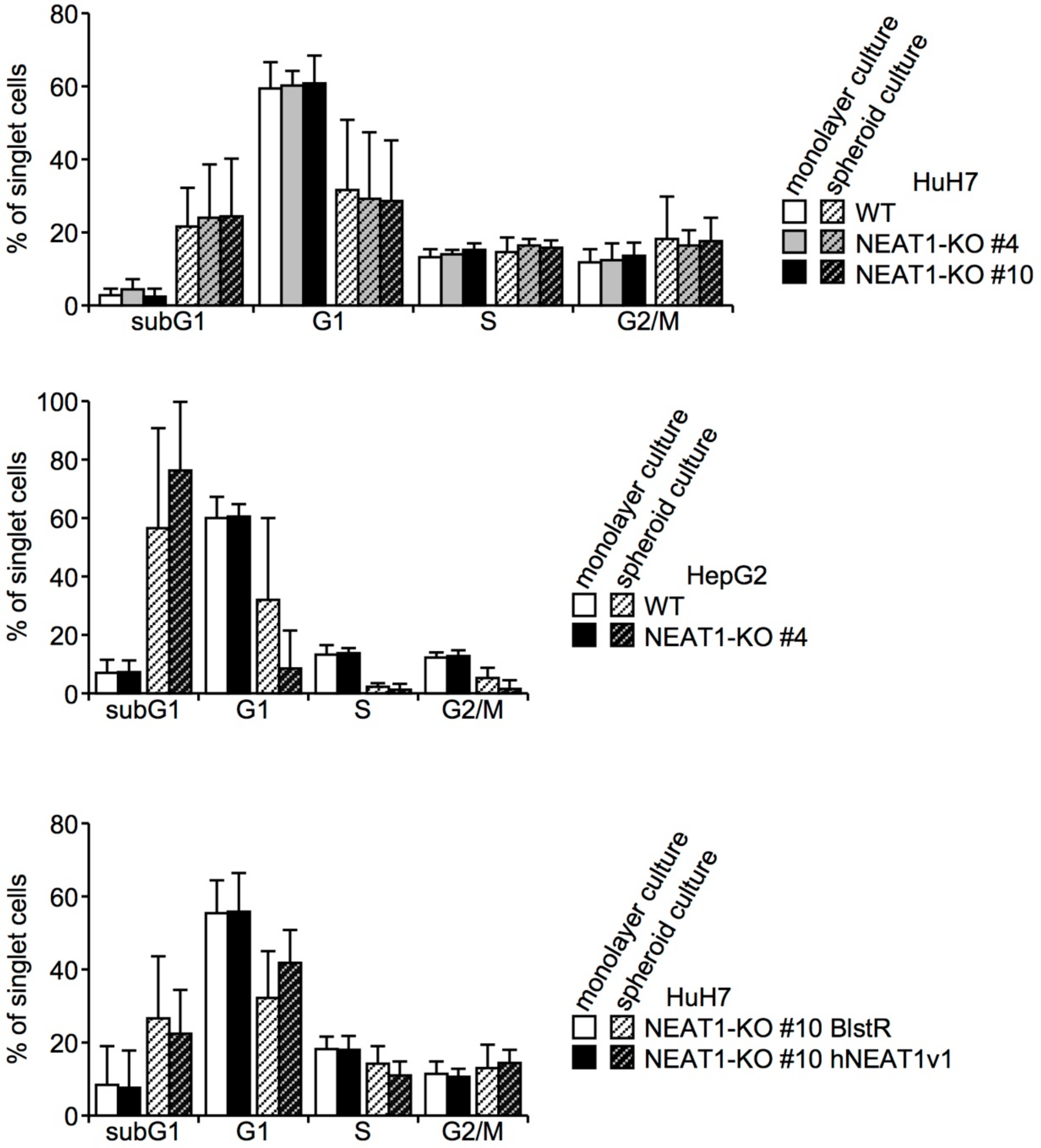

(Figure S5, continue) **B**, The cell cycle fraction of NEAT1-KO HCC cell lines. (*n* = 4–5)

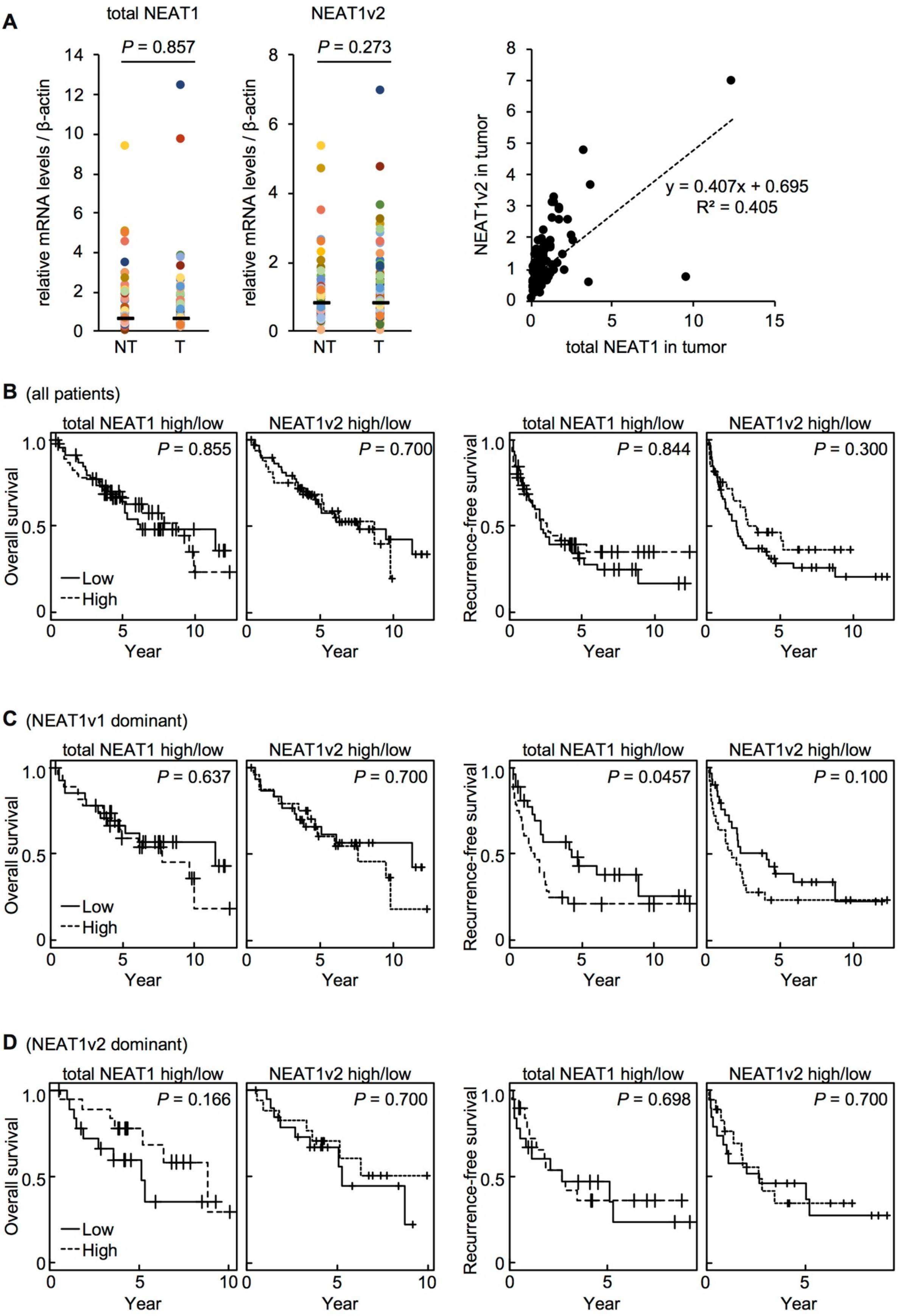

Figure S6 Relationship between NEAT1 expression and prognosis in HCC patients. **A**, (*left*) total NEAT1 and NEAT1v2 expression levels in HCC tumor (T) or non-tumor liver (NT) tissues. Bars indicate average. *P* values were calculated by Student's paired *t*-test. (*right*) Regression analysis between total NEAT1 and NEAT1v2 expression in tumor tissues. Patients above the regression line were considered as total NEAT1 dominant (NEAT1v1 dominant), while the others were NEAT1v2 dominant. **B–D**, Overall survival (*left*) and recurrence-free survival (*right*) rates of all HCC patients (**B**), NEAT1v1 dominant patients (**C**), and NEAT1v2 dominant patients (**D**) stratified according to the median values of total NEAT1 or NEAT1v2 expression. *P* values were determined by Gehan's Wilcoxon test.

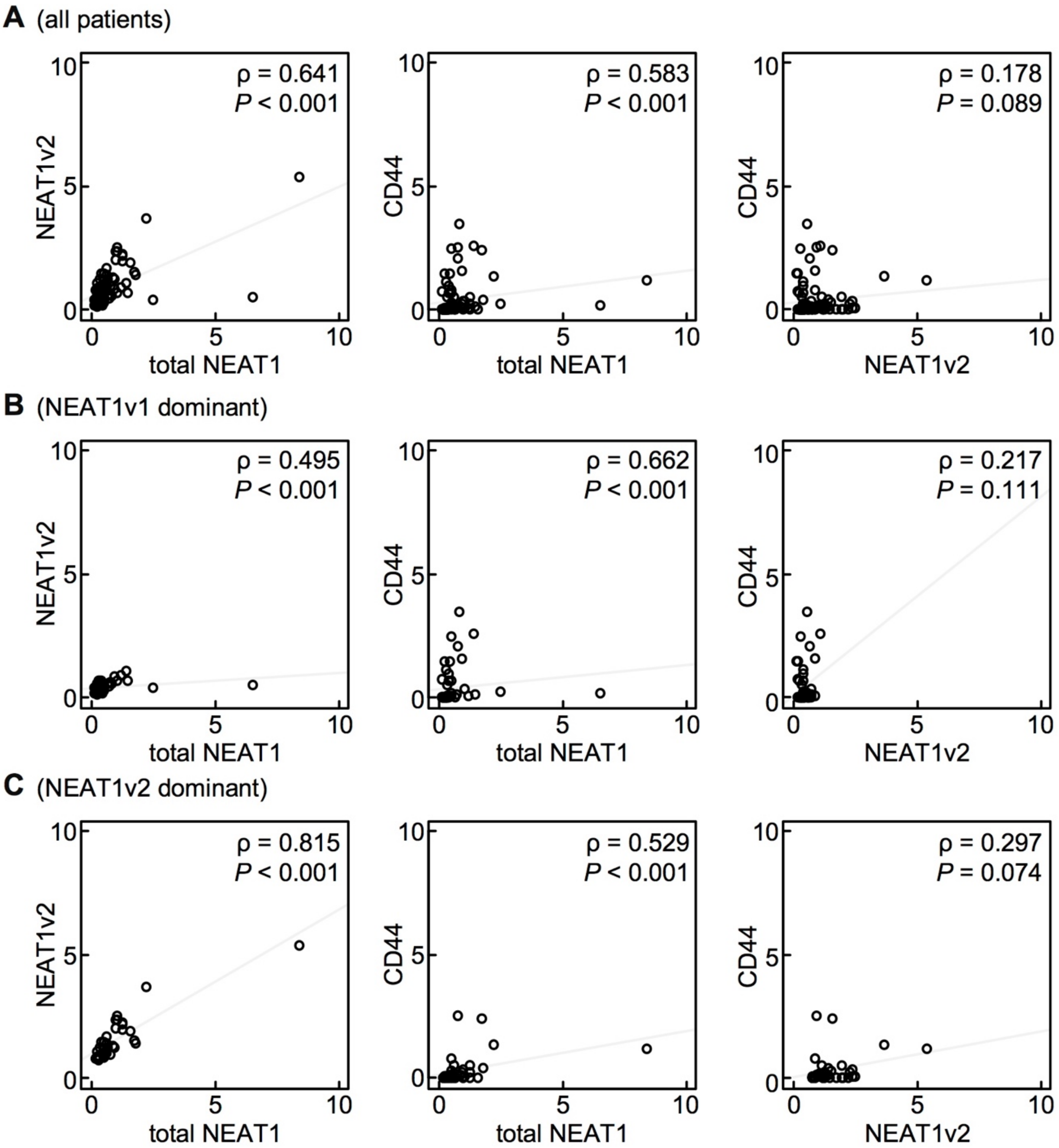

Figure S7 Correlation between CD44 and NEAT1 expression in HCC tumor tissues. **A–C**, Regression plots between NEAT1 total and NEAT1v2 (*left*), between NEAT1 total and CD44 (*middle*), and between NEAT1v2 and CD44 (*left*) in all HCC patients (**A**), NEAT1v1 dominant patients (**B**), or NEAT1v2 dominant patients (**C**) stratified according to median values of NEAT1 total or NEAT1v2 expression. *P* values were determined by Spearman's rank correlation test.
